# Mass Drug Administration of Azithromycin to Reduce Child Mortality: Only for High-Mortality Settings?

**DOI:** 10.4269/ajtmh.19-0071

**Published:** 2019-02-07

**Authors:** Kirkby D. Tickell, Emily L. Deichsel, Judd L. Walson

**Affiliations:** 1 Department of Epidemiology, University of Washington, Seattle, Washington;; 2 Department of Global Health, University of Washington, Seattle, Washington;; 3 The Childhood Acute Illness and Nutrition (CHAIN) Network, Nairobi, Kenya;; 4 Center for Vaccine Development for Global Health, University of Maryland School of Medicine, Baltimore, Maryland;; 5 Department of Medicine (Infectious Disease), University of Washington, Seattle, Washington;; 6 Department of Pediatrics, University of Washington, Seattle, Washington

Dramatic global reductions in child mortality over the past several decades have not been equitably realized across populations.^1^ Less than one quarter of sub-Saharan African countries achieved the child mortality targets outlined in the Millennium Development Goals. Without new approaches to improve child survival, these countries are also likely to fail to meet the 2030 Sustainable Development targets (less than 25 deaths before the age of 5 years per 1,000 live births).^1,2^ Biannual mass drug administration (MDA) of azithromycin has been shown to reduce child mortality in several large trials.^3–6^ Delivery via MDA offers the opportunity to impact the most marginalized and disadvantaged communities, as MDA programs appear to be among the most equitable intervention platforms in low-resource settings.^7,8^ However, in the largest of these trials (*Macrolide Oraux pour Réduire Les Décès avec un Oeil sur la Résistance* [MORDOR]), conducted in three African countries, the site-specific effect size was only significant in Niger, where the baseline mortality rate was highest. Although mortality reductions were observed in the other sites, the statistically nonsignificant effects and fact that the study was not powered to evaluate effect modification by site complicate their interpretation, especially when considering the balance of risk (including toxicity and emergence of antibiotic resistance) versus benefit. This has led to uncertainty surrounding whether such an intervention should be recommended in lower mortality settings.

In this issue of *AJTMH*, both Oron et al. and Porco et al. provide secondary analyses of available studies of azithromycin delivered by MDA to understand whether the effect size of the observed mortality benefit differs by the baseline mortality rate.^9,10^ Importantly, neither analysis concluded that there was a strong relationship between baseline mortality and effect size, although neither could exclude a modest interaction (Table 1).

**Table 1:** *P*-values for interaction between baseline mortality and the efficacy of mass azithromycin administration for reducing child mortality^9,10^

	*P*-value of interaction term*
Porco et al.	
Main analysis	0.12
MORDOR alone	0.04
Oron et al.	
DHS/MICS baseline mortality	0.02
IHME baseline mortality	0.02
MORDOR baseline mortality	0.07

DHS = Demographic and Health Surveys; MICS = Multiple Indicator Cluster Surveys; MORDOR = Macrolide Oraux pour Réduire Les Décès avec un Oeil sur la Résistance; IHME = Institute for Health Metrics and Evaluation.

* Oron et al. set a *P*-value of < 0.01 as significant and 0.01–0.1 as offering intermediate evidence for an interaction.

Although estimates from the two studies were similar, there were some discrepancies, which may be due to differences in methodology. Oron et al.^10^ modeled the effect at the individual level and tested three different sources for estimating baseline mortality using a Cox proportional hazards model. However, two of these sources of baseline mortality estimates (Demographic and Health Surveys/Multiple Indicator Cluster Surveys [DHS/MICS] and Institute of Health Metrics data) may not be representative of the population that participated in the trial. For example, the baseline mortality rates observed at the Niger site of the MORDOR study differ substantially from estimates provided by DHS/MICS. By contrast, Porco et al. estimated mortality using baseline census data from the individual studies, modeling effects using cluster-level geographical units. The use of estimates derived from the original study may raise concerns of endogeneity bias, where an included covariate (baseline mortality) is correlated with the error term of the outcome (post-intervention mortality), although the linear mixed effect model used by Porco et al. may address this concern.

If differences in baseline mortality are largely attributable to the prevention or treatment of infectious diseases, the effect of azithromycin will be dependent on the proportion of mortality attributable to pathogens that are preventable or treatable with macrolide antibiotics. This may partially explain why the MORDOR trial suggests an association with the baseline mortality. Two-thirds of the deaths in MORDOR occurred in Niger, in a setting highly endemic for malaria, for which azithromycin has demonstrated preventative efficacy.^3,11,12^ However, it is not clear how azithromycin reduces mortality, limiting our ability to base decisions regarding implementation of azithromycin MDA on the underlying potential mechanisms of benefit. Although mortality is lower in most other settings, many countries have significant capacity to benefit from reductions in child mortality. For example, Tanzania, the lowest mortality country included in these analyses, has an under-five mortality rate of 54 deaths per 1,000 live births. This is more than double the target for the Sustainable Development Goals and 10-fold higher than the average child mortality observed in high-income countries.^13^ Policy-makers are now confronted with interpreting these findings and making recommendations regarding which populations should be targeted with azithromycin to reduce child mortality.

The articles from Oron et al. and Porco et al. focus on whether differences in baseline mortality between settings can be considered to determine where such an intervention might be most effective. Although these analyses reinforce that azithromycin mass administration in high-mortality settings appears highly beneficial, they cannot exclude benefit in lower mortality settings. As the authors of both articles note, there does not need to be evidence of a relationship between baseline mortality and effect size to justify limiting mass administration of azithromycin to high-mortality settings. Targeting azithromycin delivered by MDA to high-mortality settings and populations is likely to increase the cost-effectiveness of the intervention and to minimize the risks of drug toxicity and emergence of drug resistance. Such risk stratification already forms the basis of a number of important public health interventions, including seasonal malaria chemoprevention, empiric deworming, and community management of malnutrition.

On average, it takes 17 years for novel interventions to be supported by guidelines and widely available at scale.^14^ As a result, interventions needed to accelerate progress toward the 2030 Sustainable Development targets likely need to already be supported by strong clinical trial evidence if they are to have a reasonable chance of being scaled up in time to impact these goals. Multiple trials have demonstrated that azithromycin can reduce child mortality in low-resource settings. The secondary analyses presented here do not conclusively exclude a lack of mortality benefit in lower mortality areas. As a result, decisions regarding which populations should be targeted for MDA with azithromycin should not be based on the observed statistical fluctuations in effect size from these studies. Instead, such decisions should be based on the absolute number of deaths likely to be averted by such an intervention, weighed against potential individual and population-level risks.
